# 17α-Acet­oxy-11β-hy­droxy-6α-methyl­pregn-4-ene-3,20-dione

**DOI:** 10.1107/S1600536812017631

**Published:** 2012-06-13

**Authors:** Sammer Yousuf, Saira Bano, M. Iqbal Choudhary

**Affiliations:** a H.E.J. Research Institute of Chemistry, International Center for Chemical and Biological Sciences, University of Karachi, Karachi 75270, Pakistan; b Department of Biochemistry, Faculty of Sciences, King Abdul Aziz University, Jaddah 21589, Saudi Arabia

## Abstract

The title compound, C_24_H_34_O_5_, a fungal-transformed metabolite of the injecta­ble contraceptive medroxyprogesterone acetate, consists of four fused rings (*A*, *B*, *C* and *D*; steroid labelling). Ring *A* exists in a half-chair conformation while *trans*-fused rings *B* and *C* adopt chair conformations. The five-membered ring *D* adopts an envelope conformation with the C atom bound to the methyl group at the flap. In the crystal, adjacent mol­ecules are linked by O—H⋯O and C—H⋯O hydrogen bonds, forming infinite chains along the *a* axis.

## Related literature

For biotransformational studies, see: Manosroi *et al.* (2006 ▶), Choudhary *et al.* (2005 ▶). For the crystal structures of closely related compounds, see: Yousuf *et al.* (2011 ▶, 2010 ▶). For puckering parameters, see: Cremer & Pople (1975 ▶).
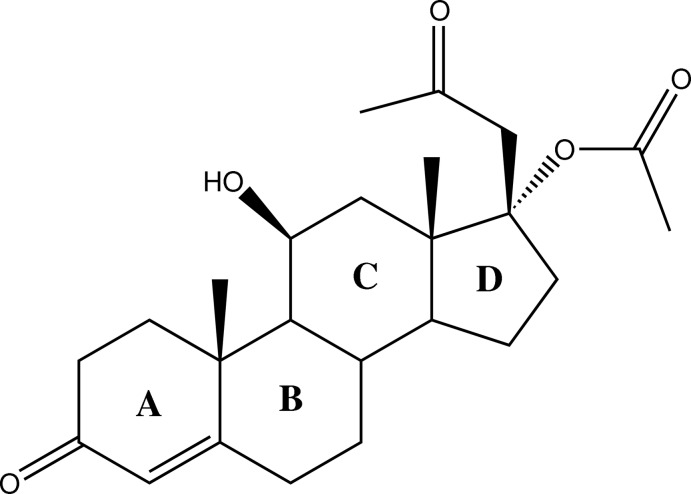


## Experimental

### 

#### Crystal data

C_24_H_34_O_5_
*M* *_r_* = 402.51
Orthorhombic, 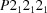

*a* = 8.2020 (6) Å
*b* = 9.8957 (8) Å
*c* = 27.972 (2) Å
*V* = 2270.3 (3) Å^3^
*Z* = 4
Mo *K*α radiation
μ = 0.08 mm^−1^
*T* = 273 K
0.33 × 0.20 × 0.16 mm

#### Data collection

Bruker SMART APEX CCD area-detector diffractometer
Absorption correction: multi-scan (*SADABS*; Bruker, 2000 ▶) *T*_min_ = 0.974, *T*_max_ = 0.987
13528 measured reflections
2431 independent reflections
1777 reflections with *I* > 2σ(*I*)
*R*_int_ = 0.059

#### Refinement

*R*[*F* ^2^ > 2σ(*F* ^2^)] = 0.043
*wR*(*F* ^2^) = 0.101
*S* = 1.01
2431 reflections
267 parameters
H-atom parameters constrained
Δρ_max_ = 0.19 e Å^−3^
Δρ_min_ = −0.14 e Å^−3^

### 

Data collection: *SMART* (Bruker, 2000 ▶); cell refinement: *SAINT* (Bruker, 2000 ▶); data reduction: *SAINT*; program(s) used to solve structure: *SHELXS97* (Sheldrick, 2008 ▶); program(s) used to refine structure: *SHELXL97* (Sheldrick, 2008 ▶); molecular graphics: *SHELXTL* (Sheldrick, 2008 ▶); software used to prepare material for publication: *SHELXTL*, *PARST* (Nardelli, 1995 ▶) and *PLATON* (Spek, 2009 ▶).

## Supplementary Material

Crystal structure: contains datablock(s) global, I. DOI: 10.1107/S1600536812017631/pv2530sup1.cif

Structure factors: contains datablock(s) I. DOI: 10.1107/S1600536812017631/pv2530Isup2.hkl

Additional supplementary materials:  crystallographic information; 3D view; checkCIF report

## Figures and Tables

**Table 1:** Hydrogen-bond geometry (Å, °)

*D*—H⋯*A*	*D*—H	H⋯*A*	*D*⋯*A*	*D*—H⋯*A*
O2—H2*A*⋯O5^i^	0.82	2.01	2.831 (3)	174
C23—H23*C*⋯O4^ii^	0.96	2.60	3.494 (5)	156

Symmetry codes: (i) 
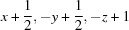
; (ii) 
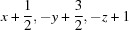
.

**Fig. 1.**
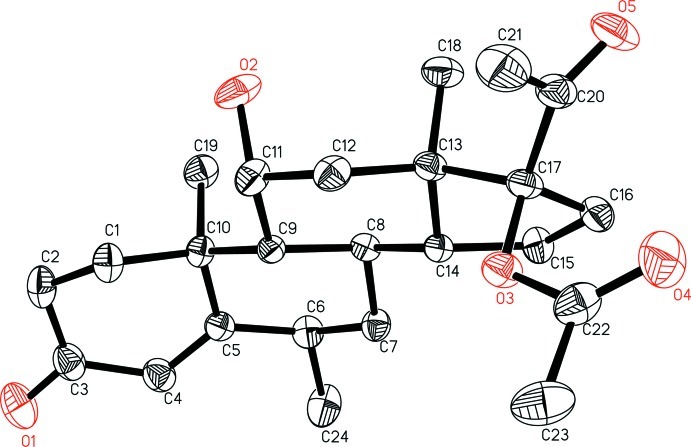
The molecular structure of the title compound with the atom numbering scheme. Displacement ellipsoids are drawn at the 30% probability level. H atoms are omitted for clarity.

**Fig. 2.**
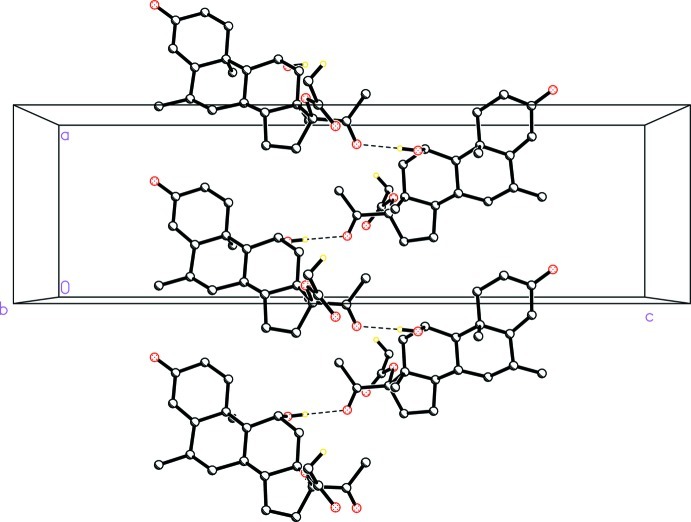
A view of the O—H···O and C—H···O hydrogen bonds (dotted lines) in the crystal structure of the title compound. H atoms non-participating in hydrogen-bonding were omitted for clarity.

## supplementary crystallographic information

### Comment

Biotransformation has been extensively applied in the production of several therapeutically important steroids on commercial scale. Such studies are done by utilizing the capability of microorganisms to convert a wide range of organic compounds into their modified derivatives and are very much useful in the production of hydoxylated metabolites (Manosroi *et al.*, 2006; Choudhary *et al.*, 2005). In the current biotransformational study of commonly used injectable contraceptive medroxyprogesterone acetate (17α-acetoxy-6α-methylpregn-4-ene-3,20-dione; MPA), was carried out by using *Cunninghamella blakesleeana* to obtain the title compound.

The title molecule (Fig. 1), is composed of four fused rings, ring A (C1–C5/C10), B (C5–C10), C (C8–C9/C11–C14) and D (C13–C17). The ring A adopts a half-chair conformation [puckering parameters (Cremer & Pople, 1975): *Q* = 0.450 (3) Å, θ = 123.4 (3)° and φ = 188.9 (5)°]. The *trans* fused rings B [*Q* = 0.535 (3) Å, θ = 172.5 (3)° and φ = 45 (3)°] and C [*Q* = 0.456 (3) Å, θ = 171.1 (3)° and φ = 84 (2)°] are in chiar conformations, whereas ring D [*Q* = 0.462 (3) Å and φ = 10.3 (4)°] adopts a C13-envelop conformation with maximum deviation of atom C13 atom from the least square plane formed by the remaining ring atoms is 0.697 (0.005) Å.

The acetyl and acetoxy substituents on C-17 exist in *pseudo equatorial* and *axial* orientations, respectively. Whereas C-11 hydroxy substituent adopts an axial orientaion. In the crystal structure, the molecules are linked by O2–H2A···O5 and C23–H23C···O4 interactions to form infinite chains running along the *a*-axix (Fig. 2, Table 1). The bond distances and bond angles in the title molecule are similar to those found in closely related compounds (Yousuf *et al.*, 2010; 2011).

### Experimental

Fungi and Culture condition:

Cultures of *Cunninghamella blakesleeana* (ATCC 9244) were grown on Sabouraud dextrose agar at 298 K and stored at 277 K. Broth media was prepared by mixing the following ingredients into distilled H_2_O (6.0 l): glucose (60.0 g), glycerol (60.0 ml), bacteriological peptone (30.0 g), yeast extract (30.0 g), KH_2_PO_4_ (30.0 g), and NaCl (30.0 g).

Fementation of medroxyprogesterone acetate:

The fungal media were transferred into 60 conical flasks (100 ml each) and autoclaved at 394 K. Seed flasks were prepared from three-day old slants of *Cunninghamella blakesleeana* (ATCC 9244) and fermentation was allowed for 4 days on a rotary shaker at 299 K. The remaining flasks were inoculated from the seed flasks. After sufficient growth of culture, medroxyprogesterone acetate (0.9 g) was dissolved in acetone (60 ml) and transferred into each flask (15 mg ml^-1^) and kept for 10 days. The culture media were filtered and extracted with dichloromethane. The extract was dried over anhydrous Na_2_SO_4_ and evaporated under reduced pressure to get brown gummy material (1.2 g) which was subjected to fractionation on silica gel column with petroleum ethe r- ethyl acetate with increasing polarity. The fraction obtained using 45% ethyl acetate in petroleum ether was finally purified by using Reversed Phase - High Performance Liquid Chromatography (RP-HPLC) (*L*-80, methanol-water 80:20 as solvent, retention time 28 min) to obtain the title compound which was recrystalized from methanol.

### Refinement

H atoms on methyl, methylene, methine and oxygen were positioned geometrically with C—H = 0.96 Å, 0.97 Å, 0.93 Å and O—H = 0.82 Å, respectively, and constrained to ride on their parent atoms with *U*_iso_(H) = 1.2*U*_eq_ (CH_2_, CH and OH) and 1.5*U*_eq_(CH_3_). A rotating group model was applied to the methyl groups. An absolute structure could not be established due to lack of anomalous dispersion effects. Therefore, 1684 Friedel pairs were merged.

### Crystal data

**Table:**

C_24_H_34_O_5_	*F*(000) = 872
*M**_r_* = 402.51	*D*_x_ = 1.178 Mg m^−^^3^
Orthorhombic, *P*2_1_2_1_2_1_	Mo *K*α radiation, λ = 0.71073 Å
Hall symbol: P 2ac 2ab	Cell parameters from 1475 reflections
*a* = 8.2020 (6) Å	θ = 2.2–19.5°
*b* = 9.8957 (8) Å	µ = 0.08 mm^−^^1^
*c* = 27.972 (2) Å	*T* = 273 K
*V* = 2270.3 (3) Å^3^	Block, colorles
*Z* = 4	0.33 × 0.20 × 0.16 mm

### Data collection

**Table:**

Bruker SMART APEX CCD area-detector diffractometer	2431 independent reflections
Radiation source: fine-focus sealed tube	1777 reflections with *I* > 2σ(*I*)
Graphite monochromator	*R*_int_ = 0.059
ω scan	θ_max_ = 25.5°, θ_min_ = 2.2°
Absorption correction: multi-scan (*SADABS*; Bruker, 2000)	*h* = −9→7
*T*_min_ = 0.974, *T*_max_ = 0.987	*k* = −11→11
13528 measured reflections	*l* = −33→32

### Refinement

**Table:**

Refinement on *F*^2^	Primary atom site location: structure-invariant direct methods
Least-squares matrix: full	Secondary atom site location: difference Fourier map
*R*[*F*^2^ > 2σ(*F*^2^)] = 0.043	Hydrogen site location: inferred from neighbouring sites
*wR*(*F*^2^) = 0.101	H-atom parameters constrained
*S* = 1.01	*w* = 1/[σ^2^(*F*_o_^2^) + (0.0475*P*)^2^ + 0.1706*P*] where *P* = (*F*_o_^2^ + 2*F*_c_^2^)/3
2431 reflections	(Δ/σ)_max_ < 0.001
267 parameters	Δρ_max_ = 0.19 e Å^−^^3^
0 restraints	Δρ_min_ = −0.14 e Å^−^^3^

### Special details

**Table:**

Geometry. All e.s.d.'s (except the e.s.d. in the dihedral angle between two l.s. planes) are estimated using the full covariance matrix. The cell e.s.d.'s are taken into account individually in the estimation of e.s.d.'s in distances, angles and torsion angles; correlations between e.s.d.'s in cell parameters are only used when they are defined by crystal symmetry. An approximate (isotropic) treatment of cell e.s.d.'s is used for estimating e.s.d.'s involving l.s. planes.
Refinement. Refinement of *F*^2^ against ALL reflections. The weighted *R*-factor *wR* and goodness of fit *S* are based on *F*^2^, conventional *R*-factors *R* are based on *F*, with *F* set to zero for negative *F*^2^. The threshold expression of *F*^2^ > σ(*F*^2^) is used only for calculating *R*-factors(gt) *etc*. and is not relevant to the choice of reflections for refinement. *R*-factors based on *F*^2^ are statistically about twice as large as those based on *F*, and *R*- factors based on ALL data will be even larger.

### Fractional atomic coordinates and isotropic or equivalent isotropic displacement parameters (Å^2^)

**Table:**

	*x*	*y*	*z*	*U*_iso_*/*U*_eq_	
O1	0.6641 (4)	0.1828 (3)	0.17149 (9)	0.0882 (10)	
O2	0.3221 (3)	0.1326 (2)	0.39265 (7)	0.0619 (7)	
H2A	0.3329	0.1258	0.4217	0.093*	
O3	0.0823 (3)	0.60806 (19)	0.42778 (7)	0.0436 (6)	
O4	−0.0792 (4)	0.7315 (3)	0.47488 (10)	0.0782 (9)	
O5	−0.1463 (4)	0.4106 (3)	0.50772 (7)	0.0625 (7)	
C1	0.5406 (4)	0.1962 (3)	0.29492 (11)	0.0468 (8)	
H1A	0.5718	0.2906	0.2967	0.056*	
H1B	0.5741	0.1532	0.3245	0.056*	
C2	0.6306 (4)	0.1302 (4)	0.25352 (12)	0.0552 (9)	
H2B	0.7467	0.1461	0.2570	0.066*	
H2C	0.6125	0.0334	0.2544	0.066*	
C3	0.5756 (5)	0.1838 (3)	0.20657 (13)	0.0537 (9)	
C4	0.4080 (4)	0.2317 (3)	0.20399 (12)	0.0498 (9)	
H4A	0.3720	0.2666	0.1750	0.060*	
C5	0.3024 (4)	0.2291 (3)	0.24025 (10)	0.0370 (7)	
C6	0.1224 (4)	0.2521 (3)	0.23329 (10)	0.0420 (8)	
H6A	0.0704	0.1634	0.2366	0.050*	
C7	0.0494 (4)	0.3411 (3)	0.27257 (9)	0.0385 (7)	
H7A	0.0860	0.4333	0.2677	0.046*	
H7B	−0.0684	0.3404	0.2694	0.046*	
C8	0.0932 (4)	0.2984 (3)	0.32326 (9)	0.0331 (7)	
H8A	0.0445	0.2099	0.3299	0.040*	
C9	0.2785 (3)	0.2875 (3)	0.32747 (9)	0.0316 (7)	
H9A	0.3188	0.3765	0.3176	0.038*	
C10	0.3551 (4)	0.1871 (3)	0.29062 (10)	0.0358 (7)	
C11	0.3415 (4)	0.2700 (3)	0.37900 (10)	0.0444 (8)	
H11A	0.4587	0.2894	0.3787	0.053*	
C12	0.2616 (4)	0.3688 (3)	0.41426 (10)	0.0414 (8)	
H12A	0.2937	0.3447	0.4465	0.050*	
H12B	0.3017	0.4593	0.4079	0.050*	
C13	0.0766 (4)	0.3693 (3)	0.41118 (10)	0.0353 (7)	
C14	0.0284 (4)	0.4012 (3)	0.35941 (9)	0.0315 (7)	
H14A	0.0796	0.4878	0.3515	0.038*	
C15	−0.1546 (4)	0.4277 (3)	0.36200 (10)	0.0436 (8)	
H15A	−0.2156	0.3438	0.3603	0.052*	
H15B	−0.1895	0.4866	0.3362	0.052*	
C16	−0.1776 (4)	0.4969 (3)	0.41121 (10)	0.0446 (8)	
H16A	−0.2088	0.5907	0.4070	0.053*	
H16B	−0.2622	0.4515	0.4294	0.053*	
C17	−0.0141 (4)	0.4875 (3)	0.43735 (10)	0.0387 (8)	
C18	0.0023 (4)	0.2353 (3)	0.42872 (11)	0.0505 (9)	
H18A	0.0294	0.1644	0.4067	0.076*	
H18B	−0.1140	0.2441	0.4307	0.076*	
H18C	0.0453	0.2138	0.4597	0.076*	
C19	0.3006 (4)	0.0393 (3)	0.29747 (12)	0.0489 (9)	
H19A	0.3279	−0.0120	0.2695	0.073*	
H19B	0.1849	0.0364	0.3025	0.073*	
H19C	0.3552	0.0015	0.3247	0.073*	
C20	−0.0259 (5)	0.4646 (3)	0.49130 (11)	0.0468 (9)	
C21	0.1183 (5)	0.4973 (4)	0.52202 (11)	0.0694 (12)	
H21A	0.0824	0.5434	0.5503	0.104*	
H21B	0.1921	0.5543	0.5046	0.104*	
H21C	0.1729	0.4152	0.5308	0.104*	
C22	0.0342 (5)	0.7246 (3)	0.44810 (12)	0.0529 (9)	
C23	0.1423 (6)	0.8383 (3)	0.43387 (14)	0.0734 (12)	
H23A	0.0804	0.9206	0.4329	0.110*	
H23B	0.1873	0.8206	0.4028	0.110*	
H23C	0.2291	0.8472	0.4567	0.110*	
C24	0.0737 (5)	0.3052 (4)	0.18406 (11)	0.0640 (11)	
H24A	0.1168	0.2466	0.1598	0.096*	
H24B	0.1167	0.3946	0.1798	0.096*	
H24C	−0.0430	0.3078	0.1816	0.096*	

### Atomic displacement parameters (Å^2^)

**Table:**

	*U*^11^	*U*^22^	*U*^33^	*U*^12^	*U*^13^	*U*^23^
O1	0.074 (2)	0.112 (2)	0.0788 (19)	0.0133 (19)	0.0404 (17)	−0.0020 (17)
O2	0.099 (2)	0.0503 (14)	0.0370 (12)	0.0260 (15)	−0.0086 (14)	0.0050 (10)
O3	0.0552 (15)	0.0343 (11)	0.0412 (12)	−0.0046 (11)	0.0072 (10)	−0.0033 (9)
O4	0.094 (2)	0.0631 (17)	0.0772 (18)	0.0083 (17)	0.0248 (17)	−0.0216 (15)
O5	0.081 (2)	0.0647 (16)	0.0420 (13)	−0.0101 (15)	0.0218 (13)	0.0063 (11)
C1	0.037 (2)	0.0492 (19)	0.054 (2)	0.0016 (18)	−0.0014 (16)	−0.0095 (16)
C2	0.038 (2)	0.056 (2)	0.071 (2)	0.0063 (17)	0.0059 (18)	−0.0132 (19)
C3	0.050 (2)	0.051 (2)	0.060 (2)	−0.0005 (19)	0.014 (2)	−0.0104 (17)
C4	0.051 (2)	0.0498 (19)	0.0481 (19)	−0.0005 (17)	0.0079 (17)	−0.0028 (16)
C5	0.043 (2)	0.0314 (15)	0.0365 (16)	0.0003 (15)	0.0038 (15)	−0.0045 (13)
C6	0.043 (2)	0.0492 (19)	0.0342 (16)	−0.0006 (16)	−0.0056 (14)	−0.0050 (14)
C7	0.0350 (19)	0.0440 (17)	0.0364 (17)	0.0029 (16)	−0.0041 (14)	−0.0025 (13)
C8	0.0358 (19)	0.0327 (15)	0.0307 (15)	−0.0036 (14)	0.0014 (13)	−0.0003 (13)
C9	0.0303 (18)	0.0312 (15)	0.0334 (15)	0.0000 (14)	−0.0012 (12)	0.0023 (13)
C10	0.0339 (19)	0.0341 (16)	0.0395 (16)	0.0014 (15)	−0.0026 (14)	−0.0046 (13)
C11	0.048 (2)	0.0449 (18)	0.0406 (17)	0.0092 (17)	−0.0057 (16)	−0.0037 (15)
C12	0.048 (2)	0.0453 (19)	0.0311 (16)	0.0030 (16)	−0.0076 (14)	−0.0013 (15)
C13	0.042 (2)	0.0348 (16)	0.0294 (15)	−0.0015 (15)	0.0043 (14)	0.0017 (13)
C14	0.033 (2)	0.0310 (14)	0.0306 (15)	−0.0008 (14)	0.0007 (13)	0.0013 (12)
C15	0.037 (2)	0.0497 (19)	0.0442 (18)	0.0005 (16)	0.0013 (16)	−0.0033 (15)
C16	0.046 (2)	0.0473 (19)	0.0404 (18)	0.0013 (18)	0.0096 (17)	−0.0001 (15)
C17	0.047 (2)	0.0360 (16)	0.0328 (17)	−0.0033 (15)	0.0062 (15)	0.0056 (13)
C18	0.066 (3)	0.0397 (18)	0.0459 (18)	−0.0063 (17)	0.0062 (17)	0.0076 (15)
C19	0.055 (2)	0.0379 (17)	0.0534 (19)	0.0024 (17)	0.0007 (18)	−0.0057 (15)
C20	0.066 (3)	0.0411 (18)	0.0338 (17)	0.0042 (19)	0.0106 (17)	0.0015 (14)
C21	0.087 (3)	0.085 (3)	0.036 (2)	0.002 (3)	−0.003 (2)	−0.0006 (19)
C22	0.074 (3)	0.0394 (19)	0.0450 (19)	0.005 (2)	−0.0062 (19)	−0.0075 (16)
C23	0.105 (4)	0.042 (2)	0.073 (3)	−0.015 (2)	−0.012 (2)	0.0018 (18)
C24	0.065 (3)	0.088 (3)	0.0385 (19)	0.023 (2)	−0.0089 (17)	−0.0116 (19)

### Geometric parameters (Å, º)

**Table:**

O1—C3	1.220 (4)	C11—H11A	0.9800
O2—C11	1.421 (4)	C12—C13	1.519 (4)
O2—H2A	0.8199	C12—H12A	0.9700
O3—C22	1.344 (4)	C12—H12B	0.9700
O3—C17	1.456 (3)	C13—C14	1.534 (4)
O4—C22	1.196 (4)	C13—C18	1.540 (4)
O5—C20	1.213 (4)	C13—C17	1.567 (4)
C1—C2	1.521 (4)	C14—C15	1.525 (4)
C1—C10	1.529 (4)	C14—H14A	0.9800
C1—H1A	0.9700	C15—C16	1.549 (4)
C1—H1B	0.9700	C15—H15A	0.9700
C2—C3	1.486 (5)	C15—H15B	0.9700
C2—H2B	0.9700	C16—C17	1.531 (4)
C2—H2C	0.9700	C16—H16A	0.9700
C3—C4	1.456 (5)	C16—H16B	0.9700
C4—C5	1.334 (4)	C17—C20	1.529 (4)
C4—H4A	0.9300	C18—H18A	0.9600
C5—C6	1.507 (4)	C18—H18B	0.9600
C5—C10	1.531 (4)	C18—H18C	0.9600
C6—C24	1.527 (4)	C19—H19A	0.9600
C6—C7	1.530 (4)	C19—H19B	0.9600
C6—H6A	0.9800	C19—H19C	0.9600
C7—C8	1.523 (4)	C20—C21	1.497 (5)
C7—H7A	0.9700	C21—H21A	0.9600
C7—H7B	0.9700	C21—H21B	0.9600
C8—C9	1.528 (4)	C21—H21C	0.9600
C8—C14	1.530 (4)	C22—C23	1.487 (5)
C8—H8A	0.9800	C23—H23A	0.9600
C9—C11	1.541 (4)	C23—H23B	0.9600
C9—C10	1.563 (4)	C23—H23C	0.9600
C9—H9A	0.9800	C24—H24A	0.9600
C10—C19	1.541 (4)	C24—H24B	0.9600
C11—C12	1.536 (4)	C24—H24C	0.9600
			
C11—O2—H2A	109.5	C12—C13—C18	112.0 (3)
C22—O3—C17	117.8 (3)	C14—C13—C18	112.1 (2)
C2—C1—C10	113.5 (3)	C12—C13—C17	116.8 (3)
C2—C1—H1A	108.9	C14—C13—C17	99.5 (2)
C10—C1—H1A	108.9	C18—C13—C17	107.8 (2)
C2—C1—H1B	108.9	C15—C14—C8	119.2 (2)
C10—C1—H1B	108.9	C15—C14—C13	104.1 (2)
H1A—C1—H1B	107.7	C8—C14—C13	113.4 (2)
C3—C2—C1	111.9 (3)	C15—C14—H14A	106.4
C3—C2—H2B	109.2	C8—C14—H14A	106.4
C1—C2—H2B	109.2	C13—C14—H14A	106.4
C3—C2—H2C	109.2	C14—C15—C16	103.8 (2)
C1—C2—H2C	109.2	C14—C15—H15A	111.0
H2B—C2—H2C	107.9	C16—C15—H15A	111.0
O1—C3—C4	121.6 (4)	C14—C15—H15B	111.0
O1—C3—C2	121.8 (3)	C16—C15—H15B	111.0
C4—C3—C2	116.5 (3)	H15A—C15—H15B	109.0
C5—C4—C3	124.7 (3)	C17—C16—C15	106.9 (3)
C5—C4—H4A	117.7	C17—C16—H16A	110.3
C3—C4—H4A	117.7	C15—C16—H16A	110.3
C4—C5—C6	122.4 (3)	C17—C16—H16B	110.3
C4—C5—C10	121.4 (3)	C15—C16—H16B	110.3
C6—C5—C10	115.9 (2)	H16A—C16—H16B	108.6
C5—C6—C24	115.1 (3)	O3—C17—C20	109.7 (2)
C5—C6—C7	112.2 (2)	O3—C17—C16	109.7 (2)
C24—C6—C7	110.3 (3)	C20—C17—C16	115.2 (3)
C5—C6—H6A	106.2	O3—C17—C13	105.5 (2)
C24—C6—H6A	106.2	C20—C17—C13	112.4 (2)
C7—C6—H6A	106.2	C16—C17—C13	103.8 (2)
C8—C7—C6	114.6 (2)	C13—C18—H18A	109.5
C8—C7—H7A	108.6	C13—C18—H18B	109.5
C6—C7—H7A	108.6	H18A—C18—H18B	109.5
C8—C7—H7B	108.6	C13—C18—H18C	109.5
C6—C7—H7B	108.6	H18A—C18—H18C	109.5
H7A—C7—H7B	107.6	H18B—C18—H18C	109.5
C7—C8—C9	109.0 (2)	C10—C19—H19A	109.5
C7—C8—C14	110.4 (2)	C10—C19—H19B	109.5
C9—C8—C14	110.0 (2)	H19A—C19—H19B	109.5
C7—C8—H8A	109.1	C10—C19—H19C	109.5
C9—C8—H8A	109.1	H19A—C19—H19C	109.5
C14—C8—H8A	109.1	H19B—C19—H19C	109.5
C8—C9—C11	114.4 (2)	O5—C20—C21	121.4 (3)
C8—C9—C10	113.2 (2)	O5—C20—C17	119.4 (3)
C11—C9—C10	114.2 (2)	C21—C20—C17	119.0 (3)
C8—C9—H9A	104.5	C20—C21—H21A	109.5
C11—C9—H9A	104.5	C20—C21—H21B	109.5
C10—C9—H9A	104.5	H21A—C21—H21B	109.5
C1—C10—C5	109.7 (3)	C20—C21—H21C	109.5
C1—C10—C19	109.5 (3)	H21A—C21—H21C	109.5
C5—C10—C19	106.8 (2)	H21B—C21—H21C	109.5
C1—C10—C9	108.1 (2)	O4—C22—O3	122.8 (3)
C5—C10—C9	108.7 (2)	O4—C22—C23	126.0 (3)
C19—C10—C9	113.9 (2)	O3—C22—C23	111.2 (3)
O2—C11—C12	112.9 (3)	C22—C23—H23A	109.5
O2—C11—C9	108.7 (2)	C22—C23—H23B	109.5
C12—C11—C9	112.7 (2)	H23A—C23—H23B	109.5
O2—C11—H11A	107.4	C22—C23—H23C	109.5
C12—C11—H11A	107.4	H23A—C23—H23C	109.5
C9—C11—H11A	107.4	H23B—C23—H23C	109.5
C13—C12—C11	113.1 (3)	C6—C24—H24A	109.5
C13—C12—H12A	109.0	C6—C24—H24B	109.5
C11—C12—H12A	109.0	H24A—C24—H24B	109.5
C13—C12—H12B	109.0	C6—C24—H24C	109.5
C11—C12—H12B	109.0	H24A—C24—H24C	109.5
H12A—C12—H12B	107.8	H24B—C24—H24C	109.5
C12—C13—C14	108.2 (2)		
			
C10—C1—C2—C3	54.1 (4)	C11—C12—C13—C14	−56.1 (3)
C1—C2—C3—O1	153.9 (4)	C11—C12—C13—C18	67.9 (3)
C1—C2—C3—C4	−28.9 (4)	C11—C12—C13—C17	−167.2 (2)
O1—C3—C4—C5	176.4 (4)	C7—C8—C14—C15	60.3 (3)
C2—C3—C4—C5	−0.8 (5)	C9—C8—C14—C15	−179.4 (3)
C3—C4—C5—C6	−166.9 (3)	C7—C8—C14—C13	−176.6 (2)
C3—C4—C5—C10	6.4 (5)	C9—C8—C14—C13	−56.2 (3)
C4—C5—C6—C24	−11.3 (5)	C12—C13—C14—C15	−168.9 (2)
C10—C5—C6—C24	175.1 (3)	C18—C13—C14—C15	67.2 (3)
C4—C5—C6—C7	−138.6 (3)	C17—C13—C14—C15	−46.5 (3)
C10—C5—C6—C7	47.9 (4)	C12—C13—C14—C8	60.0 (3)
C5—C6—C7—C8	−49.1 (4)	C18—C13—C14—C8	−63.9 (3)
C24—C6—C7—C8	−178.9 (3)	C17—C13—C14—C8	−177.6 (2)
C6—C7—C8—C9	53.6 (3)	C8—C14—C15—C16	162.3 (2)
C6—C7—C8—C14	174.5 (2)	C13—C14—C15—C16	34.7 (3)
C7—C8—C9—C11	169.6 (2)	C14—C15—C16—C17	−8.4 (3)
C14—C8—C9—C11	48.4 (3)	C22—O3—C17—C20	−56.0 (4)
C7—C8—C9—C10	−57.2 (3)	C22—O3—C17—C16	71.5 (3)
C14—C8—C9—C10	−178.4 (2)	C22—O3—C17—C13	−177.3 (3)
C2—C1—C10—C5	−47.7 (4)	C15—C16—C17—O3	92.2 (3)
C2—C1—C10—C19	69.3 (3)	C15—C16—C17—C20	−143.4 (3)
C2—C1—C10—C9	−166.1 (2)	C15—C16—C17—C13	−20.2 (3)
C4—C5—C10—C1	18.1 (4)	C12—C13—C17—O3	41.0 (3)
C6—C5—C10—C1	−168.3 (3)	C14—C13—C17—O3	−75.0 (3)
C4—C5—C10—C19	−100.6 (3)	C18—C13—C17—O3	168.0 (2)
C6—C5—C10—C19	73.0 (3)	C12—C13—C17—C20	−78.5 (3)
C4—C5—C10—C9	136.1 (3)	C14—C13—C17—C20	165.5 (3)
C6—C5—C10—C9	−50.2 (3)	C18—C13—C17—C20	48.5 (3)
C8—C9—C10—C1	174.2 (3)	C12—C13—C17—C16	156.4 (3)
C11—C9—C10—C1	−52.4 (3)	C14—C13—C17—C16	40.4 (3)
C8—C9—C10—C5	55.2 (3)	C18—C13—C17—C16	−76.6 (3)
C11—C9—C10—C5	−171.5 (3)	O3—C17—C20—O5	149.5 (3)
C8—C9—C10—C19	−63.8 (3)	C16—C17—C20—O5	25.1 (4)
C11—C9—C10—C19	69.5 (3)	C13—C17—C20—O5	−93.5 (4)
C8—C9—C11—O2	79.6 (3)	O3—C17—C20—C21	−36.3 (4)
C10—C9—C11—O2	−53.2 (4)	C16—C17—C20—C21	−160.7 (3)
C8—C9—C11—C12	−46.4 (4)	C13—C17—C20—C21	80.8 (4)
C10—C9—C11—C12	−179.1 (3)	C17—O3—C22—O4	3.1 (5)
O2—C11—C12—C13	−73.2 (3)	C17—O3—C22—C23	−178.5 (3)
C9—C11—C12—C13	50.5 (4)		

### Hydrogen-bond geometry (Å, º)

**Table:**

*D*—H···*A*	*D*—H	H···*A*	*D*···*A*	*D*—H···*A*
O2—H2*A*···O5^i^	0.82	2.01	2.831 (3)	174
C23—H23*C*···O4^ii^	0.96	2.60	3.494 (5)	156

Symmetry codes: (i) *x*+1/2, −*y*+1/2, −*z*+1; (ii) *x*+1/2, −*y*+3/2, −*z*+1.
